# Efficacy of a dual therapy based on darunavir/ritonavir and etravirine in ART-experienced patients

**DOI:** 10.7448/IAS.17.4.19787

**Published:** 2014-11-02

**Authors:** Jose Ignacio Bernardino, Francisco Xavier Zamora, Eulalia Valencia, Victoria Moreno, Jorge Vergas, Maria Jesus Tellez, Vicente Estrada, Juan Gonzalez-Garcia

**Affiliations:** 1 HIV Unit, Internal Medicine, Hospital La Paz, IdiPAZ, Madrid, Spain; 2 Servicio Enfermedades Infecciosas, Hospital Carlos III, Madrid, Spain; 3 nfectious Diseases Unit, Internal Medicine, Hospital Clinico Universitario, Madrid, Spain

## Abstract

**Introduction:**

Nucleoside reverse transcriptase inhibitors (NRTI)-sparing regimens have been studied in antiretroviral therapy (ART)-naïve patients but data with ART-experienced are scarce. NRTI-sparing regimens may be an option in patients with toxicities and for simplification reasons.

**Methods:**

Retrospective multicentre analysis including ART-experienced patients starting treatment with darunavir/ritonavir and etravirine (DRV/r 800 mg/100 mg QD or 600 mg/100 mg BID and ETV 400 mg QD or 200 mg BID) with at least six months of follow-up. Primary endpoint was proportion of patients with VL<50 copies/mL at 48 weeks with an ITT analysis (missing or switch equals failure). Secondary endpoints were safety, CD4 count and lipid changes over 48 weeks.

**Results:**

Seventy-five patients were included of whom 44 (58.6%) had HIV RNA<50 copies/mL. Baseline characteristics: median age 50 years (IQR 34–65), 72% males, 93% Caucasians, 38.6% hepatitis C, and 45.4% with CDC C stage. Median HIV duration and time on ART were 20 (IQR 7–28) and 14 years (IQR 5–21) respectively. Reasons for switching were virologic failure in 27 (36%), simplification in 25 (33.3%), toxicity in 20 (26.6%) and other 3 (4.1%). Most of them received DRV/r and ETV QD. Thirty-nine patients had NNRTI resistance mutations [28 K103N (37.3%), 6 Y181I/C (8%), 3 G190A (4%)] and 29 patients had ≥1 primary PI mutations. Main analysis (ITT) showed that 67 (89.3%) had a VL undetectable at 24 weeks (95% CI 83.1–95.5) and 57 (76%) at 48 weeks (95% CI 68.4–83.6). On treatment analysis showed that 94.3% and 89% had a viral load<50 copies at 24 and 48 weeks, respectively. 11 (14.6%) patients discontinued the regimen (three virologic failures, three switching to darunavir/ritonavir monotherapy, two to salvage regimen and three due to toxicity). No significant changes in CD4+ count and lipid changes were observed at 48 weeks.

**Conclusions:**

Dual therapy with Darunavir/ritonavir and etravirine is an efficacious and safety option in ART-experienced HIV patients even in patients on virologic failure.

